# HiCrayon reveals distinct layers of multi-state 3D chromatin organization

**DOI:** 10.1093/nargab/lqae182

**Published:** 2024-12-18

**Authors:** Ben Nolan, Hannah L Harris, Achyuth Kalluchi, Timothy E Reznicek, Christopher T Cummings, M Jordan Rowley

**Affiliations:** Department of Genetics, Cell Biology and Anatomy, University of Nebraska Medical Center, Emile St, Omaha, NE 68198, USA; Department of Genetics, Cell Biology and Anatomy, University of Nebraska Medical Center, Emile St, Omaha, NE 68198, USA; Department of Genetics, Cell Biology and Anatomy, University of Nebraska Medical Center, Emile St, Omaha, NE 68198, USA; Department of Genetics, Cell Biology and Anatomy, University of Nebraska Medical Center, Emile St, Omaha, NE 68198, USA; Department of Pediatrics, University of Nebraska Medical Center, Emile St, Omaha, NE 68198, USA; Department of Genetics, Cell Biology and Anatomy, University of Nebraska Medical Center, Emile St, Omaha, NE 68198, USA

## Abstract

Chromatin contact maps are often shown as 2D heatmaps and visually compared to 1D genomic data by simple juxtaposition. While common, this strategy is imprecise, placing the onus on the reader to align features with each other. To remedy this, we developed HiCrayon, an interactive tool that facilitates the integration of 3D chromatin organization maps and 1D datasets. This visualization method integrates data from genomic assays directly into the chromatin contact map by coloring interactions according to 1D signal. HiCrayon is implemented using R shiny and python to create a graphical user interface application, available in both web and containerized format to promote accessibility. We demonstrate the utility of HiCrayon in visualizing the effectiveness of compartment calling and the relationship between ChIP-seq and various features of chromatin organization. We also demonstrate the improved visualization of other 3D genomic phenomena, such as differences between loops associated with CTCF/cohesin versus those associated with H3K27ac. We then demonstrate HiCrayon’s visualization of organizational changes that occur during differentiation and use HiCrayon to detect compartment patterns that cannot be assigned to either A or B compartments, revealing a distinct third chromatin compartment.

## Graphical Abstract

**Graphical Abstract:**
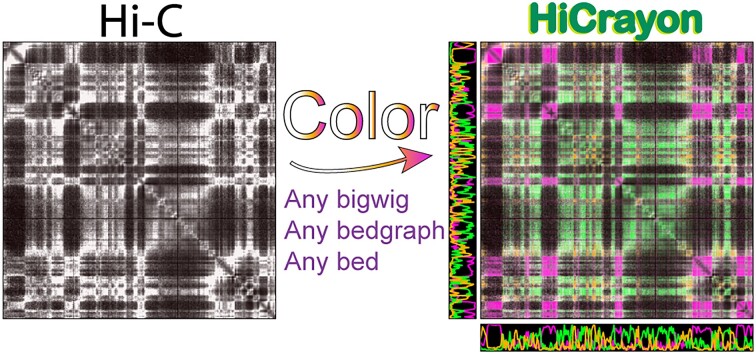

## Introduction

The study of 3D genome organization relies on integrating data from multiple sources, including the co-analysis of 2D maps of chromatin conformation with 1D genomic signals such as Chromatin immunoprecipitation sequencing (ChIP-seq) tracks for histone modifications or architectural proteins. Genome-wide chromatin conformation capture experiments, such as Hi-C, Micro-C and Pore-C, measure long-range chromatin interactions, which are most commonly represented as 2D heatmaps (1–3). These chromatin contact maps detail several features that have revealed fundamental principles of chromatin organization (4). Some of the earliest maps describe the propensity of loci to interact in A (active) and B (inactive) compartments (1), with more recent work showing that this segregation can occur at kilobase scale (5,6). Genome-wide maps of chromatin organization also reveal high-intensity punctate signals corresponding to CCCTC-binding factor (CTCF) loops in mammalian cells (7) and Polycomb (Pc) loops in *Drosophila melanogaster* (8–11).

Our understanding of 3D genome organization is often derived from the comparison of 2D chromatin contact maps with 1D genomic datasets. For example, broad-scale comparisons of the plaid-like Hi-C compartment pattern with histone modification ChIP-seq tracks reveal the propensity for chromatin with active histone modifications to locate in the A compartment (1). When viewed at fine scale, we recently showed that localization to the A compartment is a precise, fundamental characteristic of active enhancers and promoters (5). Another well-known example is the overlap between 2D punctate loops and 1D CTCF ChIP-seq data, revealing the overwhelming presence of CTCF at these loop anchors (7). In contrast, a comparison of high-intensity 2D punctate loops with 1D ChIP-seq signal revealed a lack of CTCF occupancy in *D. melanogaster* (12). Instead, *D. melanogaster* loop anchors are occupied by Polycomb (Pc) and Pipsqueak (Psq) (8–11).

Currently, the visual comparison of 2D and 1D genomic datasets at example loci is most often accomplished through the juxtaposition of heatmap and signal tracks (13,14). Indeed, there are several existing browsers and visualization tools that allow juxtaposition of 2D chromatin contacts with 1D chromatin occupancy (13–18). While useful, this simple visualization relies on ‘eyeballing’ the relationships, with the user visually aligning peaks of 1D signal with 2D features. Naturally, this current practice can be imprecise and prone to human visual biases. To aid the precise visualization of overlapping 2D and 1D features, we developed HiCrayon for the visual integration of 1D genomic signals with 2D chromatin contacts within a single matrix. We showcase the capabilities of HiCrayon by coloring chromatin contact maps by ChIP-seq signal to reveal features, including CTCF and non-CTCF associated loops, as well as the changes to chromatin organization that occur during differentiation. We also demonstrate the ability of HiCrayon to reveal distinct segregation of chromatin into multiple distinct compartment states, which the eigenvector and its commonly accepted binary ‘A’ and ‘B’ designation fails to describe. These results demonstrate the ability of HiCrayon to perform advanced data visualization to uncover fundamental principles of 3D chromatin organization.

## Materials and methods

### Data reprocessing

The lymphoblastoid Hi-C map is a combination of eight public lymphoblastoid cell lines (LCLs) deposited in ENCODE or GEO repositories as follows: AK1: ENCSR508EMN, GM12878: ENCSR410MDC, GSE63525, GM12891: ENCSR859YSL, GM12892: ENCSR075VWI, GM13976: ENCSR634FNY, GM13977: ENCSR261EVH, GM18526: ENCSR693CIM, GM19239: ENCSR264SMC. Raw fastq files were downloaded and reprocessed to align to the human genome build hg38, using juicer v1.14.08 (19) with a quality filter of 30. We made the original fastq files, individual processed hg38 .hic files, and the combined 10 billion intrachromosomal contact Hi-C LCL map (.hic) available at GEO accession GSE255264.

All Hi-C and ChIP-seq datasets used in this study are publicly available (Supplementary Table S1). The eigenvector denoting compartments was identified in each of the corresponding Hi-C datasets using POSSUMM (5). Hi-C distance normalization was performed as described previously (20) by taking the average signal at each diagonal as an expected value, in the following formula.


\begin{eqnarray*}\left( {observed + 1} \right)/\left( {expected + 1} \right)\end{eqnarray*}

### Matrix generation

HiCrayon generates a 2D matrix from any 1D signal track, such as ChIP-seq or compartment calling tracks. The resulting matrix is then weighted by contact frequencies derived from chromatin contact maps, such as a Hi-C contact matrix. HiCrayon streams contact frequencies from either a local file or URL, storing the 2D contact matrix for a user-specified region. 1D signals in bigwig or bedgraph format (i.e. ChIP-seq) or bedgraph format with positive and negative values (i.e. compartmental eigenvector) are then used to calculate a 1D to 2D signal matrix. First, 1D signal undergoes log transformation, if selected by the user, and scaling to fit all values between 0 and 1. In the case of 1D tracks that have negative values (i.e. the compartmental eigenvector), negative values are considered separately throughout the calculations and are multiplied by -1 before log transformation and scaling. Next, we create a two-dimensional signal matrix *m* where each value is the multiplicative product of the scaled 1D signal *s* in the row and column: $m[ {i,j} ] = s[ i ] \cdot s[ j ]$

If the ‘separate signal’ option is used, a user can choose a second bigwig or bedgraph signal to calculate the interactions between distinct features on chromatin, s1 and s2.


\begin{eqnarray*}m\left[ {i,j} \right] = s1\left[ i \right] \cdot s2\left[ j \right]\end{eqnarray*}

Separately, we also scale the 2D contact map between 0 and 1, after which the scaled contact matrix *c* is multiplied against the 1D signal matrix m and then converted to an 8-bit color scale to produce *h*: $h = ( {m*c} )*255$.

For the user-selected genomic interval, HiCrayon will place the 1D data on a scale from 0 to 1 according to the local minimum and maximum. This local scaling was chosen to enhance visualization of local enrichment but can result in blown out signal if there are no ‘peaks’ of signal within the user-selected region. Therefore, we suggest the default scale as a starting point and provide options for users to adjust the scale, and/or log transform the data as they see fit. Bed files can be uploaded, as a fourth column is created with 1 as the value, to simulate a bedgraph for visualization of all entries in the bed file (i.e. peak file). In addition, we include a ‘chromHMM’ option where upload of a chromHMM bed file, containing *n* states are visualized simultaneously by the RGB color in the chromHMM file itself.

Finally, matrix *h* is adjusted to the user’s desired color scheme (RGB), resulting in an RGBA value for each bin within the matrix. These calculations result in an image where the transparency is a function of the joint ChIP-seq or compartment call data and contact intensity.

### Color blending

HiCrayon allows the overlay of multiple 1D tracks on the contact matrix in order to visualize potentially overlapping features. For each 1D matrix, *m* is generated as described above. We then combine these matrices using linear interpolation, a method that determines the intermediate value within the range of discrete values. In the context of color, linear interpolation finds an intermediate between n colors. Let *Mb* be the *b*-th input matrix, where *b* = 1, 2,..., *n*, and *n* is the number of 1D signal matrices *m* in the input list. This allows the user to select *n* ChIP-seq tracks to visualize in a single matrix.

The alpha values for each matrix are then summed by ${{\alpha }_b} = \ {{M}_b}[ {:,:,3:4} ]$ with $tota{{l}_{alpha}} = \ \mathop \sum \nolimits_{b = 1}^n {{\alpha }_b}$. The blend ratios for each matrix are computed as $blend\_ratio{{s}_b} = \ {{\alpha }_b}/total\_alpha$. The color channels for each matrix are extracted as $color\_channel{{s}_b} = {{M}_b}[ {:,:,:3} ]$ and the mixed-color channels are calculated as $mixed\_color\_channels = \mathop \sum \nolimits_{b = 1}^n color\_channel{{s}_b}x\ blend\_ratio{{s}_b}$. The final alpha value is limited to the 8-bit maximum: ${{mixed}_{alpha}} = clip( {total\_alpha,\ 0,255} )$. The final mixed matrix is then obtained by stacking the mixed color channels with the mixed alpha value.

## Results

### HiCrayon enables precise visual inspection of 1D chromatin occupancy signals in 2D chromatin interaction matrices

**Figure 1.**
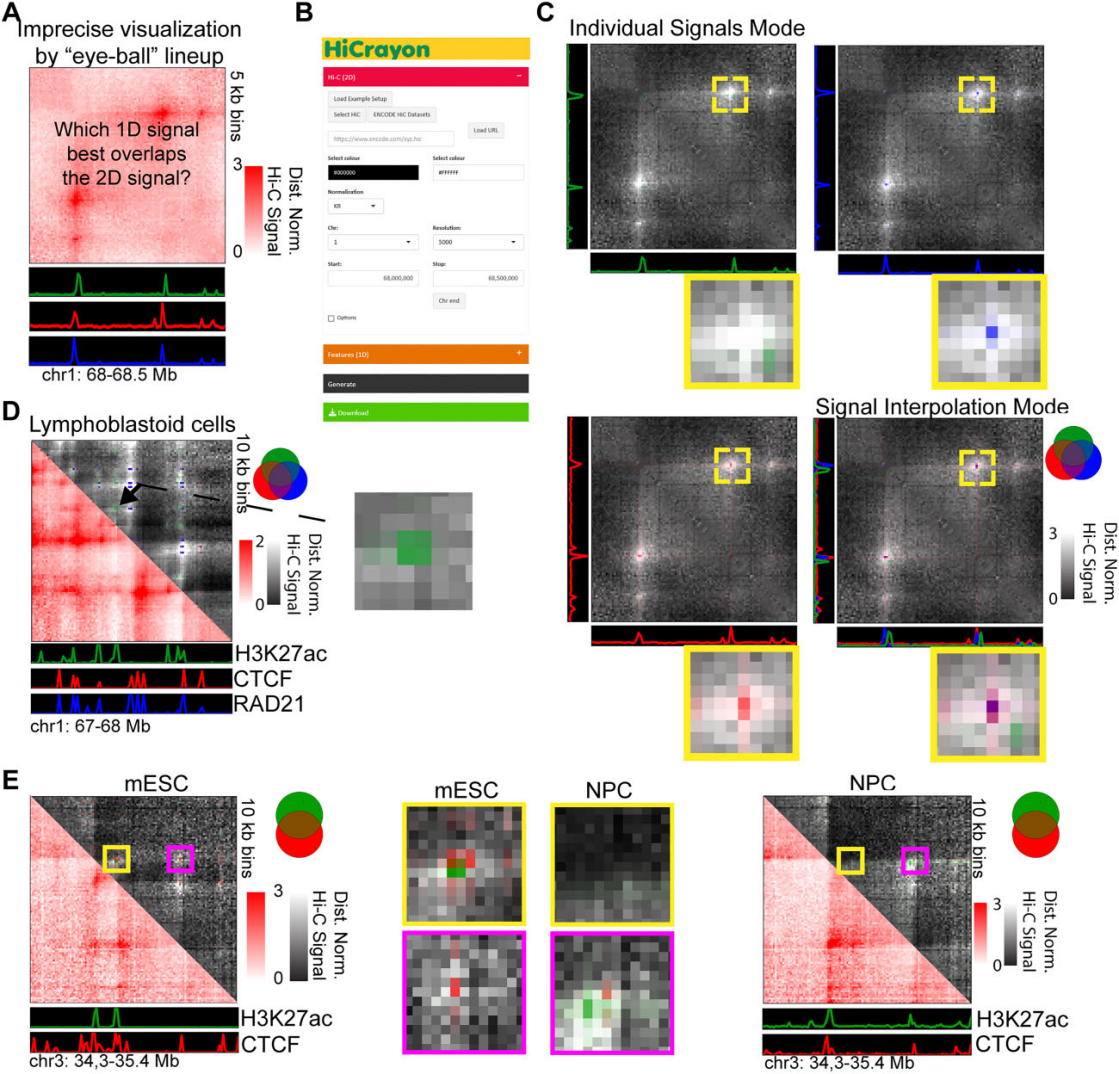
HiCrayon precisely visualizes the overlap between ChIP-seq and Hi-C signals. (**A**) A schematic demonstrating the difficulty in visually determining precise overlaps between 1D tracks (green, red, and blue) and a 2D map of chromatin contacts. (**B**) HiCrayon includes a graphical interface to color 2D maps by 1D features to visualize loci of interest. (**C**) Example of how coloring chromatin contact maps by 1D signals helps precisely determine which factors are found at the height of the chromatin interaction signal, determining that the green track is not at the peak of loop signal. Signal interpolation mode integrates the coloration of multiple 1D factors to show their overlap at interacting loci. (**D**) HiCrayon visualization of a 1 Mb region in lymphoblastoid cells and the relationship to H3K27ac (green), CTCF (red) and RAD21 (blue). Zoomed-in portion corresponds to interactions associated with H3K27ac, and not CTCF or RAD21. (**E**) Visualization of the Sox2 locus, the changes in CTCF (red), H3K27ac (green) and their associated interactions during differentiation from mESCs (left) to NPCs (right).

There are a large number of Hi-C browsers to choose from, which juxtapose 1D signals with 2D contact maps (Figure 1A) (13–18). To overcome limitations in the comparison of 1D and 2D chromatin data, we developed HiCrayon for coloring chromatin contact maps by 1D signal. HiCrayon is not meant as competition to existing browsers; instead, it should be seen as a companion tool to existing browsers, in order to pinpoint overlaps at loci of interest and create final figures. HiCrayon is implemented using a combination of Python and the R shiny package (21) for a menu-driven graphical user interface (GUI) (Figure 1B). The application is available in two forms: (i) A full-functionality, local version installed through Github, https://github.com/JRowleyLab/HiCrayon, which allows the visualization of files stored locally or via a URL, and (ii) a website version, https://jrowleylab.com/HiCrayon, with more limited functionality to allow ease of access for users who wish to visualize previously published datasets via URL, such as those stored on the ENCODE data portal (22,23). Images generated by HiCrayon can be downloaded in SVG and PNG format.

HiCrayon allows the selection of multiple 1D bigwig signal tracks to be overlayed at the selected 2D locus. Visualization is accomplished by 1D to 2D transformation of signals, weighted by the interaction matrix along with linear interpolation of color matrices (see ‘Materials and methods’ section). Advanced options allow users to adjust the degree of overlay between the 1D-to-2D signal matrix on the 2D chromatin contact matrix. Each 1D-to-2D signal matrix can be visualized separately or can be overlaid with each other within a single, fully customizable bespoke color-blended image (Figure 1C). This visualization helps distinguish 1D signals that are precisely at the high interaction sites (Figure 1C, blue and red) versus those that are proximal (Figure 1C, green). HiCrayon also allows visualization of 1D signal with negative and positive values, as commonly obtained from compartment identification (24). In this mode, HiCrayon uses two colors to distinguish positive from negative values, thereby allowing distinct visualization of A and B compartment interactions (see below).

Maps of 3D chromatin organization in mammals have revealed thousands of punctate chromatin loops (7,25,26). The most prominent loops are formed by cohesin-mediated extrusion, which is blocked at CTCF loop anchors (4,27–32). Therefore, punctate loops in mammals are typically enriched for CTCF and RAD21, a member of the cohesin complex (7,33–36). We also recently showed that CTCF loops are often comprised of fairly diffuse interactions when viewed at high resolution (5) (Supplementary Figure S1A). Using this high-resolution Hi-C map in LCLs, we colored contacts by HiCrayon to visualize the overlap between chromatin interactions and published ChIP-seq for CTCF (red), RAD21 (blue) and H3K27ac (green) (Supplementary Figure S1B). We found that HiCrayon at high resolution can help visualize the center of loop foci where an intense Hi-C signal coincides with CTCF and cohesin together (purple) (Supplementary Figure S1C and D). While the normal function of HiCrayon is to color matrices by the same 1D signal on the X and Y axes of the 2D matrix, we also include a ‘v.s.’ mode, where chromatin interactions are colored by different 1D signals on the X and Y axes, enabling visual identification of interactions where the left and right anchors are occupied by different proteins (Supplementary Figure S1E). Overall, we found that HiCrayon was able to visually distinguish interactions comprised of H3K27ac from that of CTCF/cohesin loops (Figure 1D and Supplementary Figure S1F).

We then used HiCrayon to visualize punctate loops that have been found in Hi-C maps of *D. melanogaster* Kc167 cells (8,9). In contrast to the thousands of punctate CTCF loops found in mammals, there are only a few hundred intense punctate loops in Hi-C maps of interphase *D. melanogaster* (8–10,12). While these punctate signals are commonly referred to as Polycomb (Pc) loops (9,10), the anchors are also occupied by the architectural protein Pipsqueak (Psq) (11) (Supplementary Figure S2A). Zooming in on the loop, HiCrayon captures the overlap between the loop and Pc (blue) as well as Psq (red) (Supplementary Figure S2B). In contrast, HiCrayon coloring found that, despite high H3K27me3 levels in the vicinity, these interactions precisely overlap a peak of H3K27ac (green) and not H3K27me3 (orange) (Supplementary Figure S2C). This dip in H3K27me3 and spike in H3K27ac precisely at the *D. melanogaster* Kc167 loop anchors can also be seen by the average ChIP-seq profiles across all loops (Supplementary Figure S2D) and is statistically significant (Supplementary Figure S2E), which is consistent with our previous report (11). This indicates that, despite the overlap with Pc, these Psq-associated loops correspond to islands of active H3K27ac-associated chromatin in the midst of the broader H3K27me3 repressive chromatin. Overall, we find that HiCrayon has the ability to visually identify precise overlaps between protein occupancy and features of 3D chromatin organization.

Several studies have profiled changes in genome organization that occur during differentiation (37–40). We asked if HiCrayon is able to visualize differential loops corresponding to altered occupancy at anchors in mouse embryonic stem cells (mESCs) and neural progenitor cells (NPCs) (39). Indeed, HiCrayon maps of CTCF (red) and H3K27ac (green) highlighted the altered structure of an example locus (Figure 1E). Specifically, HiCrayon reveals a loss of a short loop near the pluripotency marker Sox2, along with a gain in H3K27ac-associated interactions near the larger loop after differentiation to NPCs (Figure 1E). In each case, we found that H3K27ac was nearby but not directly at the CTCF sites (Figure 1E), an important distinction that would be missed by simple juxtaposition.

### HiCrayon enables precise visual inspection of chromatin compartments

**Figure 2.**
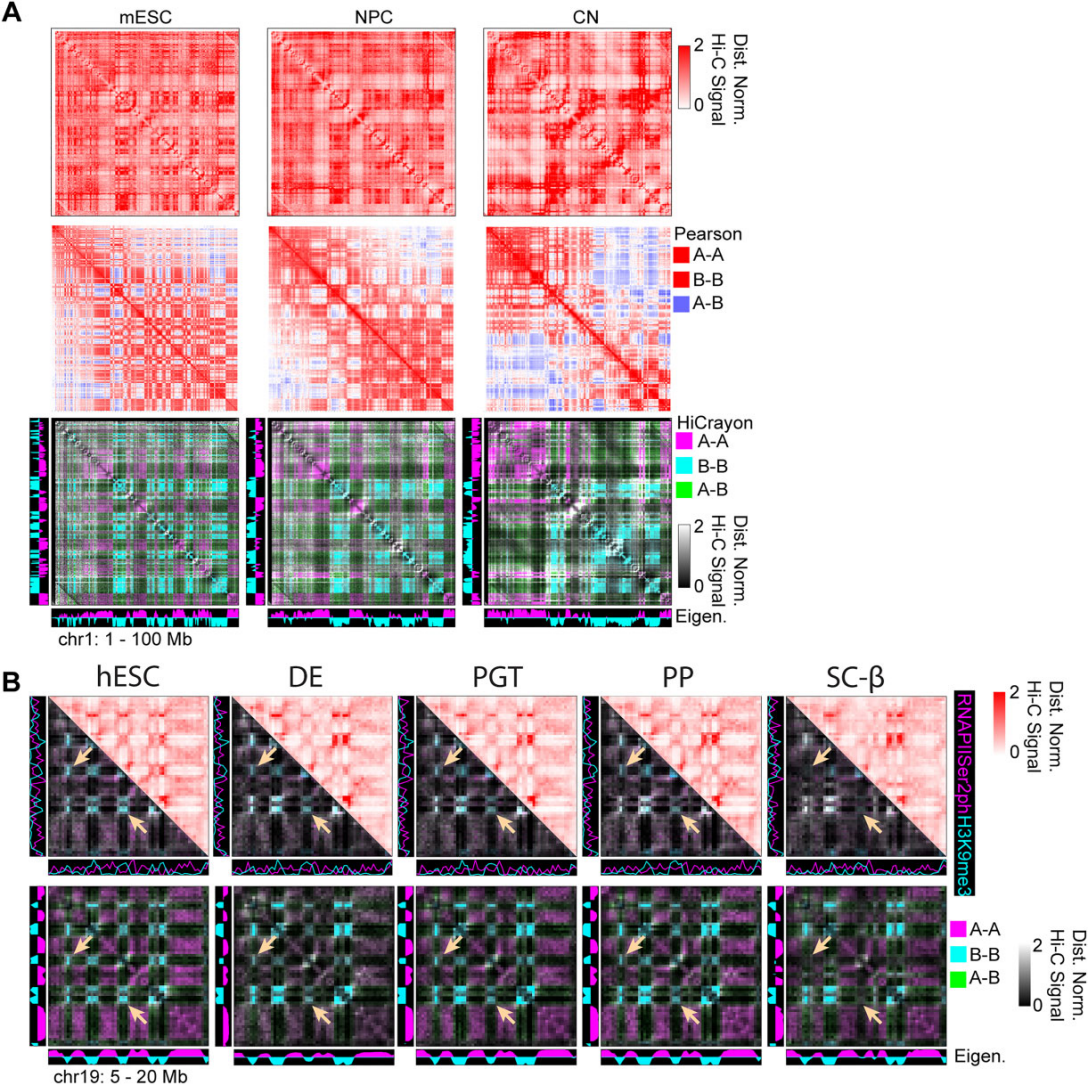
HiCrayon reveals coordinated changes in histone marks and compartments during differentiation. (**A**) Comparison of Hi-C maps, Pearson correlation matrices and HiCrayon to color compartments and thereby visualize the changes that occur during differentiation of mESCs, NPCs and cortical neurons (CNs). The color of the legend on the right indicates how features are colored in each visualization method. (**B**) Top: Hi-C and HiCrayon maps colored by RNA Polymerase Serine 2 phosphorylation (purple) and H3K9me3 (cyan) across differentiating pancreatic cells. Bottom: The same contact maps but using HiCrayon to color by the compartment eigenvector. Arrows highlight regions with decreased B–B interactions.

A and B compartments are represented by a plaid-like pattern in Hi-C maps (Figure 2A, top), and the Pearson correlation matrix is often used to visualize this pattern (Figure 2A, middle). While this strategy helps distinguish A–B interactions (blue) from A–A or B–B (red), it fails to distinguish between A–A and B–B interactions themselves, as they are both represented by the same color (Figure 2A, middle). To facilitate the distinct visualization of A–A and B–B interactions, we built into HiCrayon the ability to color the Hi-C map by the eigenvector. Coloring interactions by the eigenvector provides a visual distinction of A and B compartment interactions and highlights differences between maps (Figure 2A, bottom). Indeed, we found that HiCrayon in mESC, NPC and CN highlights the dramatic reorganization of compartments that occurs during differentiation (Figure 2A).

Recently, Hi-C maps during pancreatic islet differentiation revealed changes to chromatin organization (38). Differentiation of human embryonic stem cells (hESCs) to that of definitive endoderm (DE), primitive gut tube (PGT), pancreatic progenitor (PP) and stem cell-derived β-cells (sc-β) results in altered compartments associated with a loss of H3K9me3 at some loci (38). We used HiCrayon to color by RNAPIISer2ph (pink) and H3K9me3 (blue), which allowed direct visual identification of these changes, which corresponded to changes in H3K9me3 (Figure 2B, top). Further visualization, by coloring these maps by the eigenvector, allowed direct representation of the decreased B–B interactions precisely at loci that lose H3K9me3 during pancreatic differentiation (Figure 2B, bottom).

### HiCrayon visually distinguishes a third chromatin compartment

**Figure 3.**
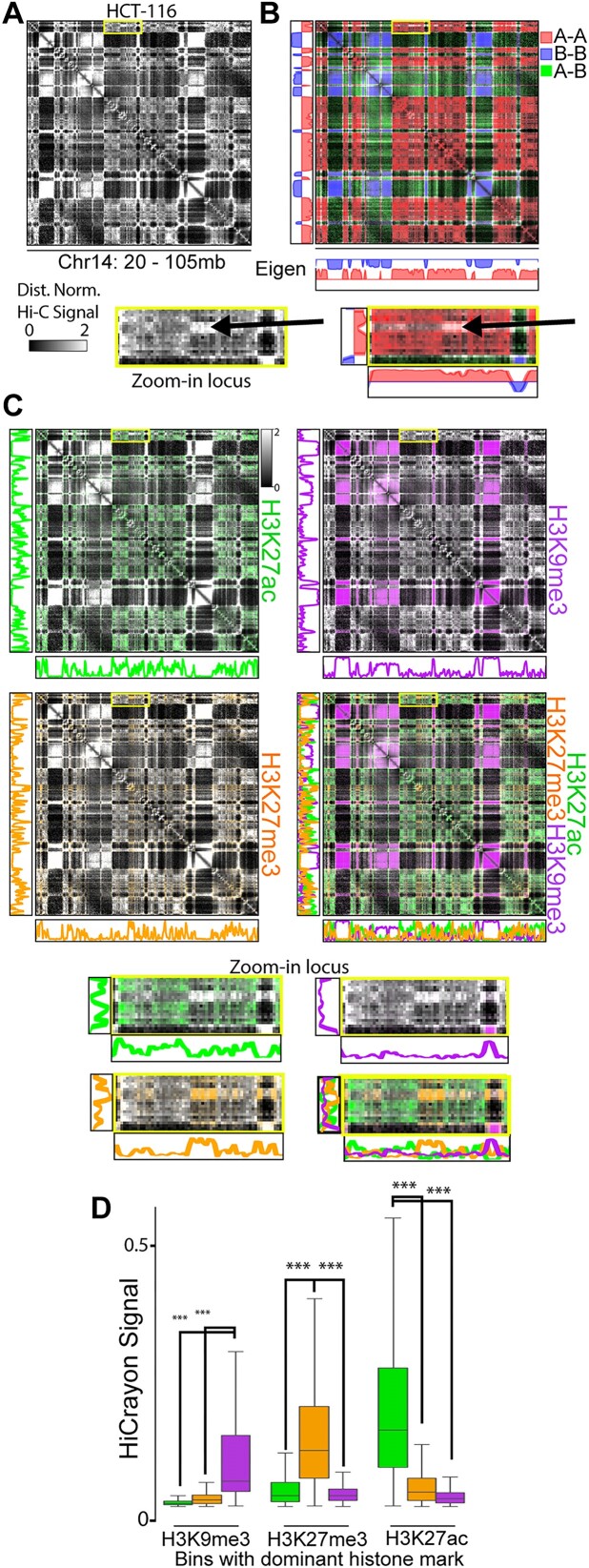
A third compartment is visually distinguishable as distinct from A or B. (**A–C**) Hi-C in HCT-116 cells showing the Hi-C signal (A), colored by the compartment eigenvector (B), or colored by H3K27ac (green), H3K9me3 (purple) and H3K27me3 (orange). Zoomed-in portion highlights a compartmental interaction with poor A/B compartment assignment and H3K27me3 associated interactions. (**D**) Quantification of the lack of overlap between marks and their associated interactions, showing the HiCrayon signal for each mark in bins with high levels of H3K9me3, H3K27me3 and H3K27ac. *** indicates *P*< .001 Wilcoxon Rank Sum test.

Annotation of A/B compartments has proven informative for numerous studies of 3D genome organization, as reviewed in (24,41). However, recent work suggests that this binary segregation represents an oversimplification and that compartments are rather more complicated than two states and may even be organized into three or more distinct compartments or subcompartments (7,42,43). Most representations of chromatin contact maps use a single gradient color scale, making it difficult to visually detect multiple states. Using HiCrayon, we provided functionality to load ChromHMM states (44), but found that the combination of binning to match Hi-C and mixing the ChromHMM-specified 15 colors only allowed visual distinction of heterochromatin from others, as revealed from picking only 2 or 3 states (Supplementary Figure S3). When we then colored interactions by the A/B compartment eigenvector, we noticed that some interactions failed to be annotated as A or B, i.e. where the eigenvector approaches zero (Figure 3A). We found that these loci have compartment-like patterns distinct from the regions that the eigenvector identified as A or B (Figure 3A, see zoom-in locus). To determine the chromatin status of these loci, we used HiCrayon to color the Hi-C map by histone modification ChIP-seq signal. While H3K27ac (green) overlaps the A–A compartment pattern, and H3K9me3 (purple) overlaps the B–B compartment pattern on this chromosome, we noticed that H3K27me3 (orange) signal is distinct from the others (Figure 3C). Indeed, we observe that interaction bins are remarkably dominant for a single histone mark, seen by a lack of color mixing when all three are overlayed (Figure 3C, bottom right). Quantification of the HiCrayon matrix shows that the interacting bins for each mark are distinct from the other marks (Figure 3D). Therefore, H3K27me3 forms distinct compartment interactions from that of H3K9me3 and H3K27ac (Figure 3A–D). These results demonstrate the ability to visually detect a more complex model of compartment organization than what is depicted by eigenvector-based compartment annotation.

## Discussion

HiCrayon represents a novel way of co-visualizing 2D and 1D ’omic datasets and is effective at detecting precise overlaps and distinguishing distinct features of genome organization. Using a simple color weighting of 2D signals by 1D features, we demonstrate the utility of HiCrayon for visualizing different aspects of chromatin organization, such as punctate loops and compartments. While this approach can reveal previously difficult-to-visualize aspects of chromatin organization, such as multi-state compartments (41,42), there are limitations. For example, due to the interpolation of 1D signal in 2D, it’s possible that HiCrayon’s default individual or interpolation modes could downplay an interaction that has ChIP-seq signal only at one anchor. To account for this possibility, HiCrayon includes an X versus Y mode, where contact maps are colored by the intersection between two distinct 1D signals.

As we demonstrate, HiCrayon visualization of the eigenvector helps distinguish A and B compartment interactions. However, it can also help identify interaction patterns that are more complex. This feature is useful both to visually evaluate the effectiveness of compartment identification (24) and to identify loci that don’t quite fit with the two-state A/B compartment model such as a third compartment or, potentially, fairly distinct subcompartments. Notably, when we color maps by marks of euchromatin, polycomb repressive chromatin and heterochromatin, we obtain a more complete picture of the predominant interacting loci. However, we also note that not all of the interactions can be colored by just those three marks, suggestive of an even more complex compartmental organization.

## Supplementary Material

lqae182_Supplemental_File

## Data Availability

A full version of HiCrayon can be downloaded and run locally from https://github.com/JRowleyLab/HiCrayon. A scaled-down web version of HiCrayon is available at https://jrowleylab.com/HiCrayon. A data accession list can be found in Supplementary Table S1. Hi-C (.hic) files for the individual and combined LCL [5] maps reprocessed to genome build hg38 are available from GSE255264. ChIP-seq in GM12878 (LCL) was used for CTCF (ENCFF232FCT), RAD21 (ENCFF822QJA) and H3K27ac (ENCFF087YCU). The high resolution Hi-C maps of *D. melanogaster* Kc167 cells are available from GSE80702 [8] and GSE89112 [10]. Hi-C files for mouse genome build mm10 ES, NPC and CN [39] are available from GSE161259. Hi-C for human genome build hg38 pancreatic differentiation [38] are available from GSE210524. Hi-C in HCT116 genome build hg38 is available from ENCFF573OPJ, with ChIP-seq available in hg38 for HCT116 in H3K27ac (ENCFF277XII), H3K27me3 (ENCFF232QSG) and H3K9me3 (ENCFF572IBD). The bedgraph chromHMM file for HCT116 in genome build hg38 is available at ENCFF897EAK. Code is available at Zenodo https://doi.org/10.5281/zenodo.14082776.
