# Supplementary material for: HiCrayon reveals distinct layers of multi-state 3D chromatin organization
Source: NAR Genom Bioinform. 2024 Dec 18;6(4):lqae182. doi: 10.1093/nargab/lqae182 (PMC11655295; doi:10.1093/nargab/lqae182)
Supplement: lqae182_Supplemental_File [file lqae182_supplemental_file.docx]

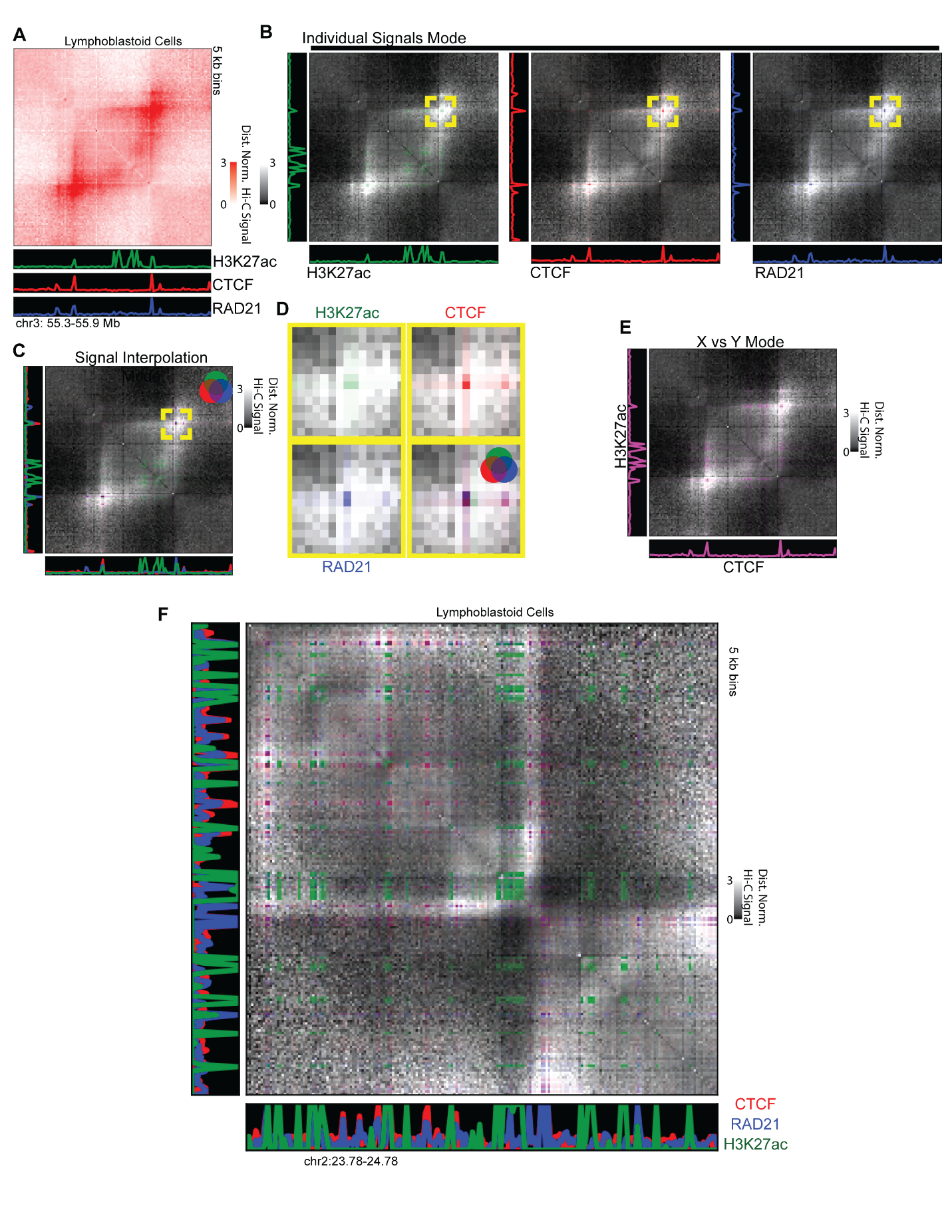
Supplementary Figure 1. A-D) Hi-C map (A) showing a punctate loop in lymphoblastoid cells with ChIP-seq signals and HiCrayon coloring (B) for H3K27ac (green), CTCF (red), and RAD21 (blue), along with interpolation of all three (C) with a zoomed-in view of the loop (D). E) Visualization of interactions using “X v.s. Y” mode, where CTCF is at one anchor and H3K27ac is at the other. F) A larger view of interactions associated with CTCF, RAD21, and/or H3K27ac.


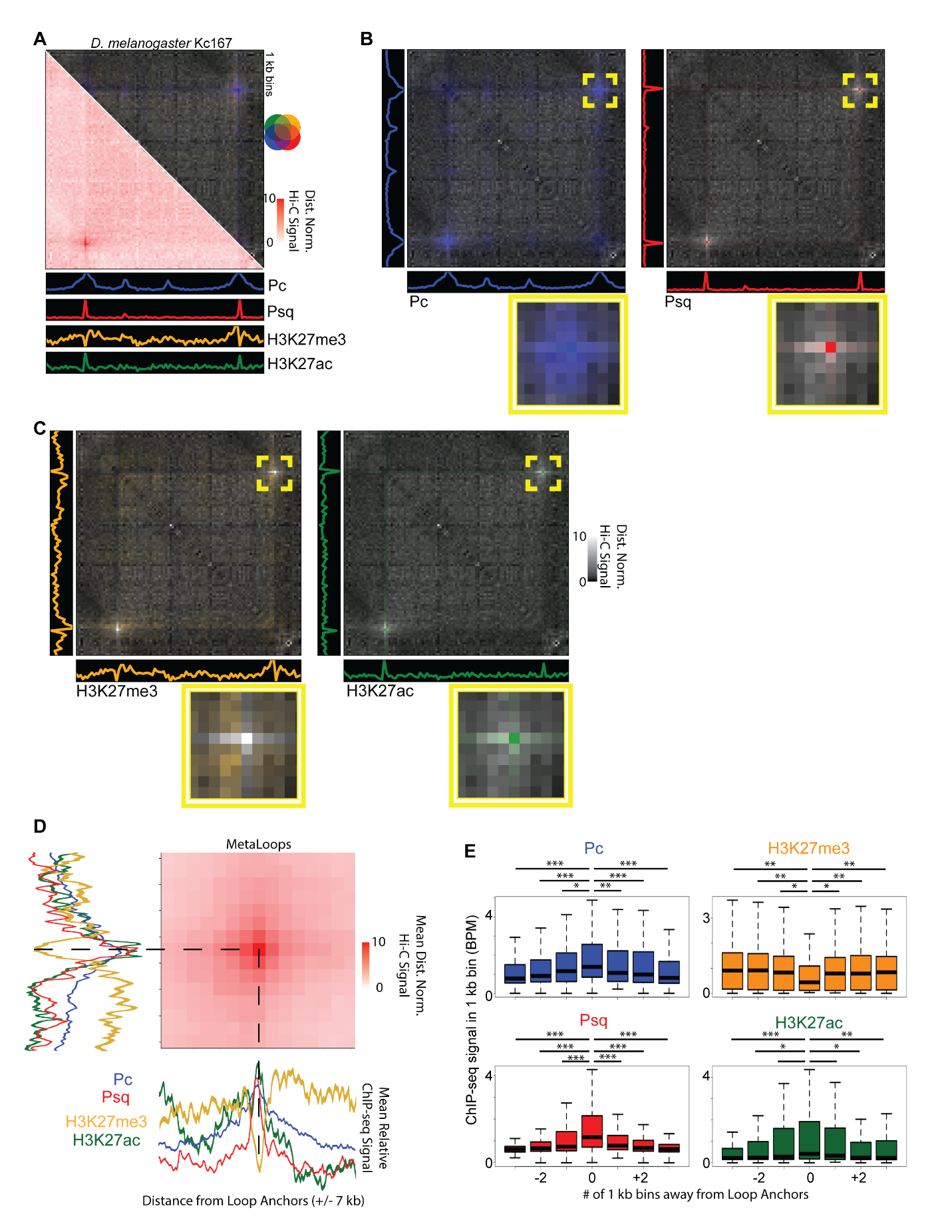


Supplementary Figure 2. A) HiC (bottom left) and HiCrayon (top right) map in D. melanogaster Kc167 cells with ChIP-seq for Pc (blue), Psq (red), H3K27me3 (orange), and H3K27ac (green). B&C) The same loop with a zoomed in view of the loop colored by each. D) Average Hi-C signal (Metaloops) across loops, along with the average ChIP-seq signal for Pc (blue), Psq (red), H3K27me3 (orange), and H3K27ac (green). E) Boxplots showing the distribution of ChIP-seq signal at the center of the loop as well as one, two, and three bins away. * p<0.05, ** p<0.01, ** p<0.001 Kolmogorov-Smirnov test v.s. the center signal.


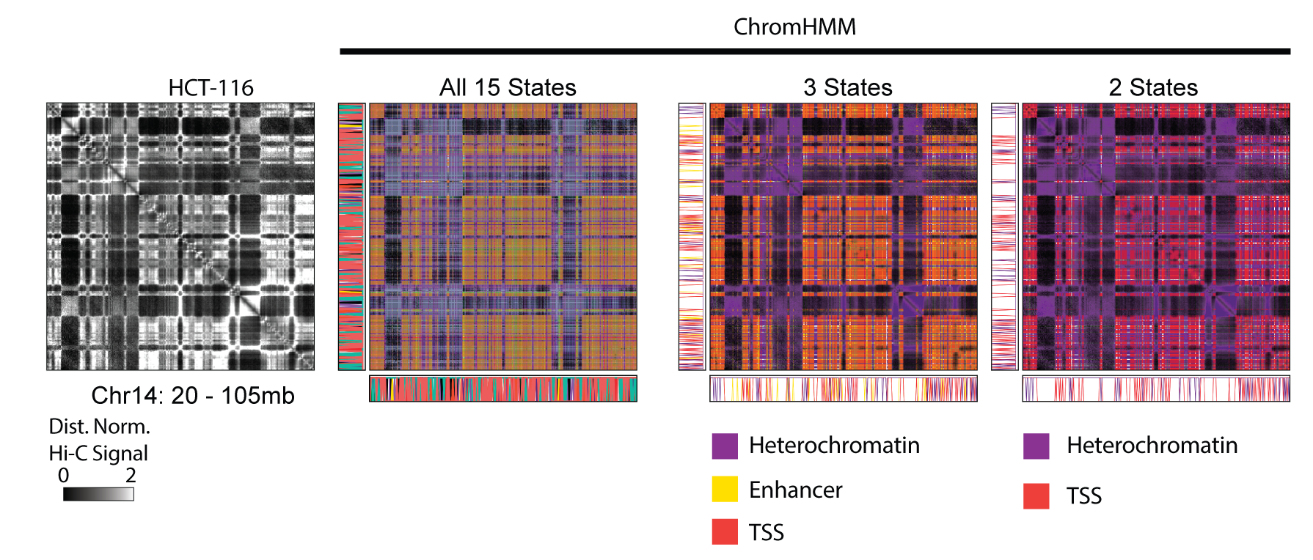


Supplementary Figure 3. HiC and HiCrayon maps in HCT116 cells colored by the 15 state ChromHMM model, as well as three or two selected states. Coloring is designated by ChromHMM color codes.

| **Data Type** | **Origin** | **IP** | **Genome** | **Accession** |
| --- | --- | --- | --- | --- |
| Hi-C | LCL | NA | hg38 | GSE255264 |
| ChIP-seq | LCL | RAD21 | hg38 | ENCFF822QJA |
| ChIP-seq | LCL | CTCF | hg38 | ENCFF232FCT |
| ChIP-seq | LCL | H3K27ac | hg38 | ENCFF087YCU |
| Hi-C | Kc167 | NA | dm6 | GSE80702+GSE89112 |
| ChIP-seq | Kc167 | H3K27ac | dm6 | GSE80702+GSE89112 |
| ChIP-seq | Kc167 | H3K27me3 | dm6 | GSE80702+GSE89112 |
| ChIP-seq | Kc167 | Psq | dm6 | GSE80702+GSE89112 |
| ChIP-seq | Kc167 | Pc | dm6 | GSE80702+GSE89112 |
| Hi-C | ES | NA | mm10 | GSE161259 |
| Hi-C | NPC | NA | mm10 | GSE161259 |
| Hi-C | CN | NA | mm10 | GSE161259 |
| Hi-C | hESCs | NA | hg38 | GSE210524 |
| Hi-C | DE | NA | hg38 | GSE210524 |
| Hi-C | PGT | NA | hg38 | GSE210524 |
| Hi-C | PP | NA | hg38 | GSE210524 |
| Hi-C | SC-beta | NA | hg38 | GSE210524 |
| ChIP-seq | hESCs | RNAPIISer2ph | hg38 | GSE211101 |
| ChIP-seq | hESCs | H3K9me3 | hg38 | GSE211101 |
| ChIP-seq | DE | RNAPIISer2ph | hg38 | GSE211101 |
| ChIP-seq | DE | H3K9me3 | hg38 | GSE211101 |
| ChIP-seq | PGT | RNAPIISer2ph | hg38 | GSE211101 |
| ChIP-seq | PGT | H3K9me3 | hg38 | GSE211101 |
| ChIP-seq | PP | RNAPIISer2ph | hg38 | GSE211101 |
| ChIP-seq | PP | H3K9me3 | hg38 | GSE211101 |
| ChIP-seq | SC-beta | RNAPIISer2ph | hg38 | GSE211101 |
| ChIP-seq | SC-beta | H3K9me3 | hg38 | GSE211101 |
| Hi-C | HCT116 | NA | hg38 | ENCFF573OPJ |
| ChIP-seq | HCT116 | H3K27ac | hg38 | ENCFF277XII |
| ChIP-seq | HCT116 | H3K27me3 | hg38 | ENCFF232QSG |
| ChIP-seq | HCT116 | H3K9me3 | hg38 | ENCFF572IBD |
| chromHMM | HCT116 | NA | hg38 | ENCFF897EAK |

Supplementary Table 1. Datasets and accessions used in this study.
